# Prevalence of hepatitis B and C, and syphilis among aspirant migrant workers of Bangladesh

**DOI:** 10.4314/ahs.v23i2.17

**Published:** 2023-06

**Authors:** Jinia Afroz, Fatema Tuz Jubyda, Sanzida Sharmin, Masud Rana, Amit Kumar Dey, Tasmia Farzana, Murshed Hasan Sarkar

**Affiliations:** 1 Department of Microbiology, Primeasia University, Dhaka, Bangladesh; 2 Department of Microbiology, Jahangirnagar University, Savar, Bangladesh; 3 Bangladesh Council of Scientific and Industrial Research (BCSIR), Rajshahi, Bangladesh

**Keywords:** Aspirant migrant workers, Hepatitis B and C, syphilis

## Abstract

**Background:**

In Bangladesh, labour migration is a source of employment and workers' remittances are critical to poverty mitigation. The aim of this study was to assess the prevalence of hepatitis B, C, HIV, tuberculosis, syphilis, kidney and liver diseases along with presence of infections among aspirant migrant workers of Bangladesh.

**Method:**

This study was carried out from September-December 2019. We analysed data collected on screening tests of specific diseases of aspirant workers. For each test, the prevalence was computed with 95% confidence interval. Association between categorical data was determined by the Chi-square test.

**Results:**

A total of 2385 aspirants, 1988 (83.35%) males, aged between 18 and 65 years (29.76±6.578) were studied. Positive results for screening tests of HBsAg were 38 (1.6%,), anti-HCV were 2 (0.08%), TPHA were 25 (1.05%) and VDRL were 5 (0.21%) though no individual was positive for HIV and TB. Elevated level of SGOT (n=99, 4.2%), SGPT (n=322, 13.5%), RBS (n=57, 2.4%), bilirubin (n=46, 1.92%), creatinine (n=7, 0.3%) and ESR (n=19, 0.8%) were found in the workers.

**Conclusion:**

Diagnosis of diseases of workers is obligatory before going abroad to safeguard the health of the workers and residents of destination country. Consequently, it will contribute to reducing the global burden of infectious diseases.

## Introduction

International migration is a long-established and universal phenomenon of today's globalised world and is critical for the economic growth of many nations^1^. There are many reasons for migration to another country including work, education, family reunification and fleeing from disasters and dispute^2^. In 2017, some 258 million international migrants were estimated globally, with 80 million inhabiting in Asia^3^. By recent estimates, there will be approximately 1.4 billion new economically active people in low income countries^4^ by 2050, of whom around 40% will hardly find meaningful employment in their countries^5^.

In Bangladesh, like many other countries of origin, migration of labour is a source of employment, and workers' remittances are critical to poverty mitigation and for counterbalance the foreign trade deficit^6^. Almost 3 million Bangladeshi household members earned their livelihood abroad in 2011 according to the report of National Population and Housing Census^7^. In 2014, some 426,000 people migrated to another country on temporary labour contracts^7^.

Nations approved medical and diagnostic centres are particularly important for governing the medical exams of aspirant migrant workers; and certified health providers in countries of origin carry out those medical exams. These tests are mandatory as certificate of medical fitness is necessary for being able to work abroad. Aspirant migrant workers should have reported the following tests in their pre-departure health assessments: complete physical examination, HBV, HCV, HIV, tuberculosis, syphilis, blood grouping, diabetes tests, and pregnancy test along with tests for presence of any infections. Migrant workers must not be at risk of having HBV, HCV, HIV, tuberculosis, syphilis or pregnancy.

Hepatitis B virus (HBV) infection, a major cause of morbidity and mortality globally was responsible for chronic infection of 248 million people in 2010 and approximately 686,000 deaths were occurred due to complications associated with HBV infections^8^-^9^ in 2013. In Bangladesh, data on the burden of chronic HBV infection are limited; however, previous small-scale studies had assessed the prevalence of HBsAg, a marker of chronic HBV infection, to be 3–7% among the general population and 1.5-12% among children aged < 5 years^10^-^12^. Limited data had been found about the prevalence of hepatitis C virus (HCV) in Bangladesh where studies were carried out on limited population subjected to screening for the presence of HCV; however, studies reported that the prevalence of HCV^13^ among Bangladeshi immigrants in Spain was 0.09% and that in the UK was 0.6%.

A report showed that approximately 13,000 adults and children were living with HIV in Bangladesh^14^. HIV-prevalence among the general population was low (<0.1%) in Bangladesh, but it was remarkably high among at-risk populations such as sex workers, injecting drug users and men who have sex with men^15^. A recent report showed migrant workers lacked adequate knowledge of modes of HIV/AIDS transmission^16^.

The prevalence of syphilis was moderately high (5.7 %) in the population of Bangladesh^17^. Among street-based sex workers, however, a high prevalence of syphilis infection (32.6%) has been reported^18^.

In the face of the tuberculosis (TB) case-load in migrant populations, there is ongoing discussion about what will be the best way to identify TB in migrant populations^19^. Every year, 300,000 people in Bangladesh develop active TB, putting an enormous burden on the economy and the health system.^20^

The burden of chronic kidney disease (CKD) is higher low-income countries, particularly in Asia, which also experienced a change in the disease burden from infectious diseases to chronic illnesses 21-22. Bangladesh holds the record of rising annual prevalence of CKD or renal insufficiency^23^.

Aspirant migrant workers must not be at risk of having HBV, HCV, HIV, tuberculosis, syphilis or been pregnant. The aim of this study was to assess the prevalence of hepatitis B and C, HIV, tuberculosis, syphilis, kidney and liver diseases along with presence of any infections among aspirant migrant workers of Bangladesh. This screening was obligatory to certify the medical fitness required to be eligible for working abroad and to establish the measures necessary to safeguard the migrant workers and residents of the destination country.

## Methods

The present work was a cross-sectional retrospective study where we analysed data collected at the Allied Diagnostics Ltd in Dhaka, Bangladesh during the period September-December 2019. This diagnostic centre received 2385 aspirant migrant workers of whom 1988 were males (83.35%); and aged between 18 and 65 years (29.76±6.578). Aspirant migrant workers with symptoms or with a positive disease history were not included in this study. Each worker was examined by the centre's medical staff after receiving informed consent. They were asked to undergo different mandatory medical tests for visa application. Test results were communicated to aspirant migrants and positive cases were recognized as unfit for the visa processing. The institutional authority in charge of the diagnostic Centre gave approval to carry out the data collection and use of these data anonymously for scientific aims. Ethical approval was not required because the study was based on data routinely collected for visa processing and stored according to the Bangladeshi law of privacy.

Hepatitis B virus surface antigen (HBsAg), hepatitis C virus antibody (anti-HCV) and antibodies to both HIV 1 and 2 (anti-HIV) were detected by enzyme-linked immunosorbent assay (ELISA) by using EVOLIS Twin Plus System (Bio-Rad Laboratories, USA).

Syphilis screening was carried out by using the Venereal Disease Research Laboratory (VDRL) test (Omega Diagnostic, UK) and the *Treponema pallidum* hemagglutination assay (TPHA) test (Omega Diagnostic, UK).

For tuberculosis (TB) diagnosis, chest X-Ray (CXR) was done. If CXR was suggestive of TB along with the presence of signs and symptoms of TB, acid-fast bacilli (AFB) sputum microscopy and culture were done.

All positive screened cases for any diagnosis were referred to public hospitals for treatment.

The liver enzymes such as serum glutamic-oxaloacetic transaminase (SGOT), serum glutamic-pyruvic transaminase (SGPT) and liver metabolites bilirubin were analysed in the serum samples of the applicants. These tests were performed using Dimension EXL-200 analyser following the manufacturer's instructions under standard conditions. The level of SGOT below 50 U/L and 45 U/L and were considered normal for male and female respectively. The level of SGPT below 56 U/L was considered normal for both male and female. Higher level of SGOT/ SGPT than the designed normal level indicated liver damage. The level of bilirubin below 1.2mg/dL was considered normal whereas, above >1.2mg/dL was considered different types of liver or bile duct problems.

Biochemical tests such as random blood sugar (RBS) (normal level, <140mg/dL) and creatinine (normal level, <1.4 mg/dL for male, <1.2 mg/dL for female) levels were measured using Dimension EXL-200 analyser following manufacturer's instruction. Higher level of RBS indicated diabetic condition. Elevated level of creatinine was considered as a sign of kidney disease. Hematological tests such as level of hemoglobin was measured using Orphee Mythic 22 AL analyser and erythrocyte sedimentation rate (ESR) (normal level, <20mm/hour for male, <29mm/hour for female) was measured using Ves-Matic Cube 30 analyser. Elevated level of ESR was considered as the presence of any infection.

Pregnancy tests were performed for female aspirants using pregnancy urine test strips.

### Statistical analysis

Descriptive statistics were used to assess demographic characteristics and screening tests results by Microsoft excel spreadsheet. Results were also expressed as mean and standard deviation (Mean ± SD). For each screening test, the prevalence was computed with 95% confidence interval (95% CI) and association between categorical data was determined by the Chi-square test. A two tailed p-value, <0.05 was considered statistically significant.

## Results

A total of 2385 aspirant migrant workers of whom 1988 were males (83.35%); aged between 18 and 65 years (29.76 ± 6.578; average age =29.76; standard deviation, SD=6.578), were studied.

Of 2385 workers, 772 individuals (32.37%) were in age group of 18-25, where 741 (95.98%) male and 31 (4.02%) female. Table 1 shows study population by sex and their age-group. In age-group 26-35, among 1150 individuals (48.22%), 864 (75.13%) were male and 286 (24.87 %) females. in age-group 36-45, among 408 aspirants (17.11 %), 332 (81.37%) were male and 76 (18.63 %) females. In age-group 46-55, among 49 (2.06%) aspirants, 45 (91.84 %) were male and 4 (8.16 %) females. In age-group 56-65, among 6 (0.25%) aspirants, all were male (6, 100%) with no female candidates (Table 1).

**Table 1:** Study population by sex and their age-group (No. =2385)

	Total		
	
Age group	N (%)	Male (%)	Female (%)
18-25	772 (32.37)	741 (95.98)	31 (4.02)
26-35	1150 (48.22)	864 (75.13)	286 (24.87)
36-45	408 (17.11)	332 (81.37)	76 (18.63)
46-55	49 (2.06)	45 (91.84)	4 (8.16)
56-65	6 (0.25)	6 (100)	0 (0)

Total	2385	1988 (83.35%)	397 (16.65%)

Of 2385 aspirants, 38 (1.6%) and 2 (0.08%) were HBsAg and anti-HCV positive respectively. Among 38 individuals (1.6%), 33 males (1.66%; 95% CI =0.01-0.02) and 5 female (1.26%, 95% CI =0.0003-0.004, p>0.05) were HBsAg positive. Among 2 anti-HCV positive individuals (0.08%), 1 was male (0.05%, 95% CI =0-0.001) and 1 (0.25%, 95% CI =0-0.0012, p>0.05) was female.

There was no significant average age difference between seropositive and sero-negative HBsAg, being respectively 28.71 years (SD = 6.08) and 29.78 years (SD = 6.58; p >.05), while for anti-HCV positive and negative individuals, average age was respectively 33 years (SD = 14.14) and 29.76 years (SD = 6.53; p > .05).

Table 2 shows the distribution of different diagnosis tests results per sex along with p-value.

**Table 2:** Distribution of different diagnosis test results per sex (No. = 2385)

Diagnosis	Male (M)		Female (F)		P-value

	Positive, n (%)	Negative, n (%)	Positive, n (%)	Negative, n (%)	
HBsAg	33 (1.66)	1955 (98.34)	5 (1.26)	392 (78.74)	0.56
Anti-HCV	1 (0.05)	1987 (99.95)	1 (0.25)	396 (99.75)	0.2
HCV RNA	1 (0.05)	1987 (99.95)	1 (0.25)	396 (99.75)	0.2
HIV I & II	0 (0)	1988 (100)	0 (0)	397 (100)	-
VDRL	3 (0.15)	1985 (99.85)	2 (0.5)	395 (99.5)	0.16
TPHA	22 (1.11)	1966 (98.89)	3 (0.76)	394 (99.24)	0.53
TB	0, 0 (0, 0)	1988 (100)	0, 0 (0,0)	397 (100)	-
AST/SGOT (>50 U/L for M, >45 U/L for F)	87 (4.38)	1901 (95.62)	12 (3.02)	385 (96.98)	0.23
ALT/SGPT (>56 U/L	301 (15.14)	1687 (84.86)	21 (5.30)	376 (94.71)	1.5
Bilirubin (>1.2mg/dL)	44 (2.21)	1944 (97.79)	2 (0.5)	395 (99.5)	0.02
Random Blood sugar (RBS) (>140mg/dL)	44 (2.21)	1944 (97.79)	13 (3.27)	384 (96.73)	0.21
Creatinine (>1.4 mg/dL for M, >1.2 mg/dL for F)	5 (0.25)	1983 (99.75)	2 (0.5)	395 (99.5)	0.39
Haemoglobin (>18 g/dL, <10g/dl)	0, 0 (0, 0)	1988 (100)	0, 0 (0,0)	397 (100)	-
ESR (>20mm/hr for M, >29mm/hr for F)	11 (0.55)	1977 (99.45)	8 (2.02)	389 (97.98)	0.002
Urine pregnancy test (PT)	-	-	6 (1.51)	391 (98.49)	-
Unfit	203 (10.21)	1785 (89.79)	36 (9.07)	361 (90.93)	0.48

Of 2385 workers, none were positive for HIV and TB. TPHA was positive in 25 subjects (1.05%), 22 males (1.11%; 95% CI =0.005-0.013), 3 female (0.76%; 95% CI =0-0.003, p>0.05), whereas 5 individuals (0.21%) of 2385 tested were positive to the VDRL test, 3 of them were male (1.5%; 95% CI =0-0.003), 2 were female (0.5%; 95% CI =0-0.002, p>0.05). Average age for TPHA positive subjects was 26.8 years (SD = 5), not significantly different to TPHA -negatives (average age = 29.79, SD = 6.6; p > .05). Average age for VDRL positive was 25 years (SD = 4.9), not significantly different to VDRL-negatives (average age = 29.78, SD = 6.6; p > .05).

The liver enzymes, SGOT was found in elevated level (>50 U/L for male, >45 U/L for female) in 99 (4.2%) workers, 87 (4.38%; 95% CI =0.03-0.04) were male and 12 (3.02%; 95% CI =0.002-0.008, p>0.05) were female. Their average age was 29.71 years (SD = 6.2), not significantly different to subjects having normal level of SGOT (29.76; SD = 6.6; p >.05). SGPT was found in higher level (>56 U/L) in 322 (13.5%) aspirants, 301 (15.14%; 95% CI =0.11-0.14) were male and 21 (15.14%; 95% CI =0.005-0.013, p>0.05) were female. Their average age was 30.79 years (SD = 6.4), not significantly different to subjects having normal level of SGPT (29.6; SD = 6.6; p >.05). Bilirubin was found in higher level (>1.2mg/dL) in 46 (1.92%) workers. The proportion of having elevated level of bilirubin was higher in males (44, 2.21%; 95% CI = 0.01-0.02) than in females (2. 0.5%; 95% CI = 0-0.002; p = 0.02). Their average age was 26.54 years (SD = 5.6), not significantly different to subjects having normal level of bilirubin (29.84; SD = 6.7; p >.05). No significant association was found between the presence of HBsAg (HBsAg positive) and elevated level of SGOT, SGPT or bilirubin (p >.05).

Of 397 female workers, 6 (1.51%) were found to be pregnant. Average age for pregnant women was 28.67 years (SD = 2.2), but there was no significant difference between ages of pregnant female workers and those who were not [(31.44; SD = 6.8); p >.05).

Elevated level of RBS (>140mg/dL) that created diabetic condition was found in 57 (2.4%) workers, 44 (2.21%; 95% CI =0.01-0.02) male and 13 (3.27%; 95% CI =0.002-0.008, p>0.05) female. Their average age was 34.12 years (SD = 7.3), not significantly different to subjects having normal level of RBS (29.35; SD = 6.5; p >.05).

Elevated level of creatinine (>1.4 mg/dL for male, >1.2 mg/dL for female) that indicated kidney damage was found in 7 (0.3%) workers, 5 (0.25%; 95% CI =0-0.004) male and 2 (0.5%; 95% CI =0-0.002, p>0.05) female. Their average age was 34 years (SD = 5), not significantly different to subjects having normal level of creatinine (29.75; SD = 6.7; p >.05).

Higher level of ESR (>20mm/hour for male, >29mm/ hour for female), indicated the presence of any infections, was found in 19 (0.8%) workers. The proportion of having elevated level of ESR was higher in males (11, 0.55%; 95% CI =0-0.01) than in females (8, 2.02%; 95% CI =0.001-0.006, p=0.002). Their average age was 32.63 years (SD = 4.72), not significantly different to workers having normal level of ESR (29.73; SD = 6.6; p >.05).

Table 3 shows sex was the significant predictor of liver diseases and occurrence of infections (p<0.05).

**Table 3:** Bivariate association between sex and results of the screening tests

Suspected disease	Male versus Female		

	OR	CI	P-value
Hepatitis B	1.3	0.51-3.42	0.56
Hepatitis C	0.2	0.01-3.2	0.2
Syphilis	0.92	0.35-2.42	0.86
Kidney disease	0.5	0.10-2.58	0.39
Liver disease	2.63	1.14-6.07	0.01
Diabetes	0.67	0.36-1.3	0.2
Other infections	0.27	0.11-0.68	0.002

OR= Odd Ratio, CI= Confidence Interval (95%)

For visa approval, subjects who fulfilled the criteria to be fit were approved. Of 2395 aspirant workers, 239 (10%) were unfit after screening tests, 203 (10.21%, 95% CI =0.07-0.1) were male and 36 (9.07%, 95% CI =0.01-0.02, p>0.05) were female. Their average age was 29.83 years (SD = 6.67), not significantly different to subjects who were fit (29.75; SD = 6.57; p >.05).

For all screening tests performed, no statistically significant differences were found for average age of subjects. The sex of the subjects was statistically significant only for occurrence of infections (p=0.002) and liver diseases (p=0.01).

## Discussion

Fair and safe labour migration is one of the major concerns since people are breaking their local boundaries and migrating to different places and countries. Bangladesh is one of the major labour sending countries of the world. Each year a large number of people migrate to different countries for both long- and short-term employment on their own accord from Bangladesh^24^. It creates employment, ensures stability to foreign exchange reserve of the country. For safe and smooth migration, aspirant migrant workers need to go through mandatory medical examinations prior to departure according to the rules and regulations. In this study, we assessed the prevalence of hepatitis B and C, HIV, tuberculosis, syphilis, kidney and liver diseases along with presence of infections among aspirant workers of Bangladesh though results might not be completely indicative because of inadequate sample size. Moreover, some studies depict that a small portion of virus carrying persons do not show any symptoms at all^25^. In this study, serological markers of HBV, HCV and syphilis were determined in asymptomatic aspirant migrant individuals who were tested in a certified referral hospital in order to get medical certification.

In our study on 2385 workers, 2.77% had serological markers of past or active infections where 1.58% were positive for HBsAg, 0.08% for anti-HCV, 1.05% for VDRL and 0.21% were positive for TPHA. No subject was found to carry both HBsAg/anti-HCV. Those data were consistent with another study conducted by Huda et al, who showed the overall seroprevalence rate of HBV was 1.42% and HCV was 0.10% among all blood donors during 2007 to 2011 in Bangladesh^26^. Another study conducted by Hasan Ashraf *et al.* in Bangladesh also showed 0.7% participants were found positive for HBsAg and 0.2% was positive for anti-HCV^11^.

From the study of Mamun-Al-Mahtab et al, it was found that 0.88% tested positive for anti-HCV among 1018 individuals of different age groups and sex which was consistent with the results of our study^12^. The present study revealed that the prevalence of HBV markers was higher in males (1.38%) than in females (0.21%). This finding is consistent with the study conducted by Mamun-Al-Mahtab *et al.*, found that the prevalence of HBV markers was higher in males than in females^12^; however, the present study had not found any statistically significant differences of the prevalence of HBV marker as per sex differences.

Biochemical tests were important to be performed for evaluation of liver condition since viral infection had several different clinical manifestations 27. In our study a significant proportion of HBsAg and anti-HCV carriers were asymptomatic, with elevated level of SGOT, SGPT and bilirubin in serum. Rahman *et al*, conducted a study on 59,227 patients with liver diseases where they found majority of those patients were males (67.9%) 28. In our study it was also found that males were more likely to have liver diseases than females.

Prevalence of syphilis was found to be 32.60% in street based, 57% were in brothel based female sex workers (FSWs) in Dhaka, Bangladesh18. This result is comparable with our neighbouring country, where FSWs were found 24.2% and 22.9% syphilis- positive in the Ahmedabad and Surat in India^29^. Another study stated 56.7% street based FSWs were infected with two or more pathogens of sexually transmitted diseases in Rajshahi, Bangladesh^30^. But the prevalence of syphilis among aspirant migrant workers in Bangladesh was not well documented. The present study showed 1.26% workers had syphilis. There was no co-infection observed among syphilis (TPHA/VDRL) positive and hepatitis (HBsAg) positive individuals.

Some limitations might affect our observations, such as the sample size and sensitivity of diagnostic kit. Moreover, it was not possible to check vaccination history because maximum of aspirant workers did not have any documentation.

## Conclusion

Our study has been carried out during a 4-month period with 2385 aspirant migrant workers undergone different diagnostic screening tests in Bangladesh. Although the conclusions drawn from the reported subjects could be partially limited, a significant proportion (10.21%) of aspirant workers presented with at least one condition that made them unfit for working abroad. Therefore, testing and improving knowledge of serologic status of aspirant migrant workers substantially help the managing of infectious hazard in destination country.

## Abbreviations

HBsAg: Hepatitis B virus surface antigen
anti-HCV: Hepatitis C virus antibody
VDRL: Venereal Disease Research Laboratory
TPHA: Treponema pallidum hemagglutination assay
TB: Tuberculosis
SGOT: Serum glutamic-oxaloacetic transaminase
SGPT: Serum glutamic-pyruvic transaminase
RBS: Random blood sugar
ESR: Erythrocyte sedimentation rate
